# From photonic technologies to microfluidics—A review on the techniques which revolutionize liquid biopsy, opening a new era in cancer therapy

**DOI:** 10.1002/hsr2.70147

**Published:** 2024-10-28

**Authors:** Subham Sarkar, Soubhagya Ghosh, Samraggi Chakraborty, Jenifer Rajak, Ekanansha R. Chowdhury, Arup K. Mitra, Ajoy Kumer, Bikram Dhara

**Affiliations:** ^1^ Postgraduate and Research Department of Biotechnology St. Xavier's College (Autonomous) Kolkata India; ^2^ Postgraduate and Research Department of Microbiology St. Xavier's College (Autonomous) Kolkata India; ^3^ S.N. Pradhan Centre for Neuroscience, Taraknath Palit Siksha Prangan University of Calcutta Kolkata India; ^4^ Department of Biotechnology University of Engineering and Management Kolkata India; ^5^ Department of Chemistry IUBAT‐International University of Business Agriculture & Technology Dhaka Bangladesh; ^6^ Centre for Global Health Research Saveetha Institute of Medical and Technical Sciences, Saveetha Medical College and Hospital Chennai India; ^7^ Department of Health Sciences Novel Global Community and Educational Foundation Sydney New South Wales Australia

**Keywords:** cancer biomarkers, liquid biopsy, microfluidics, photonic technologies

## Abstract

**Background and Aims:**

Cancer therapy is one of the most researched upon medical field in the world. Non invasive technologies such as liquid biopsy are gaining more importance in cancer therapy because of their manifold advantages over traditional invasive biopsy methods. Liquid biopsy is used to analyze nucleic acids such as ctDNA, cfDNA and RNA, cellular and subcellular components such as proteins, extracellular vesicles and circulating tumor cells in various biological fluids such as blood, urine, cerebrospinal fluid, pleural fluid and ascites fluid for diagnosis of cancer.

**Methods:**

Liquid biopsy has a wide range of applications such as assessment of residual diseases and tumors which cannot be biopsied easily and prediction of CAR‐T response and response to immune checkpoint inhibitors. It can also be used to know the efficacy of cancer drugs in a patient by analyzing multiple samples. Liquid biopsy is becoming more popular as it allows biopsy of those samples in which solid tumor biopsies are challenging or impracticable.

**Techniques and Results:**

To achieve comprehensive insight on the status of cancer in a patient, various cutting edge liquid biopsy techniques have been developed. Microfluidics and photonic technologies, along with PCR, next generation sequencing, advanced and innovative molecular and cell biology approaches and imaging techniques have expanded the domain of liquid biopsy and elevated the accuracy of liquid biopsy results.

**Conclusion:**

This review discusses about the contributions of some widely used methods along with microfluidics and photonic technologies in detection of cancer biomarkers by liquid biopsy.

## 1 INTRODUCTION

A liquid biopsy, also known as fluid biopsy or fluid phase biopsy, is the analysis of non‐solid biological tissue, primarily blood.^1^ The term “Liquid biopsy” was coined for the first time by Catherine Alix‐Panabières and Klaus Pantel. Like traditional biopsy, this type of technique is mainly used as a diagnostic and monitoring tool for diseases such as cancer, with the added benefit of being largely noninvasive.^2^ Unlike a biopsy, liquid biopsies do not test tumor tissue directly. Instead, they test for evidence of a tumor. There is no guarantee that the evidence of a tumor will be detectable in a single blood sample. Liquid Biopsy is performed when an individual has advanced cancer that is metastatic cancer which spreads from the original tumor site breaking off into tumor pieces and flows through the bloodstream.^2^ A liquid biopsy test involves a simple blood draw. A medical assistant will take a blood sample for laboratory testing. In the lab, Blood separation centrifuges will separate the blood cells from the plasma. A specialist trained to analyze fluid and tissue samples for signs of disease, will look for ctDNA in the plasma. ctDNA can identify the presence of residual cancer cells after surgery and monitors cancer relapse after successful treatment. Circulating tumor DNA (ctDNA) refers to DNA released by cancerous cells into the blood stream. Scientists can purify and analyze ctDNA using next‐generation sequencing (NGS) or PCR‐based methods such as digital PCR. A liquid biopsy assay may provide an alternative and assist in identifying key genetic alterations that aid in diagnosing, treating, and monitoring periods of relapse and remission in cancer patients.^3^ Additionally, a liquid biopsy assay is easily repeated and may provide indications of ongoing metastasis and insight into the aggressive disease state. Furthermore, liquid biopsy methods offers clinicians valuable insights into the dynamic nature of cancer and guiding personalized therapeutic strategies.^4^ This dynamic monitoring is particularly crucial in the era of precision medicine, where treatments are tailored to individual patients based on the specific genetic mutations driving their cancer. Its noninvasive nature and ability to capture disease dynamics make it a versatile tool with far‐reaching implications for healthcare.^5^ Food and Drug Administration‐approved liquid biopsies such as CTC tests allow medical assistants to predict diagnosis and monitor one's condition. ctDNA tests can identify genetic errors in cancer cell DNA.

## 2 DIFFERENT BIOMARKERS USED IN LIPID BIOPSY

Biomarkers are defined as biological molecules found in blood, other body fluids, or tissues that are a sign of a normal or abnormal process or a condition or disease. A biomarker may be used to see how well the body responds to a treatment for a disease or condition. Liquid biopsy is a technique linked to biomarkers. Unlike traditional tissue biopsy techniques, lipid biopsy has a lesser risk of complications, thanks to its noninvasive nature.^6^ Additionally, the liquid biopsy also possesses additional advantages like speed, safety, etc. In this process, body fluids are analysed for the detection of cancer‐specific biomarkers. Thus it has emerged as a promising way for diagnosis and management of cancer patients. It is a key player in guiding treatment decisions such as assessing changes or progressions guiding treatment decisions and determining suitable therapeutic regimens.^7^

The most common biomarkers used in lipid biopsy are as follows:

a) *CTCs*: The biomarkers usually considered for liquid biopsy are called CTCs (circulating tumour cells). These are cells that originate in the primary tumour and circulate in the body fluids. Their concentration is deficient in blood, approximately one in a million leukocytes, and their presence is usually associated with lower response rates and shorter overall survival in patients.^8^ Different enrichment, isolation, and identification methods have been developed based on their physical and biological characteristics.^9^ The most common method to determine CTC count in whole blood is epithelial cell adhesion molecule (EpCAM) but it can miss cells with stem‐like characteristics. Another sequestration technique is laser capture microdissection which involves encapsulating a CTC on hydrogel and sequencing it after laser extraction.^8^
b) *Circulating tumour DNA (ctDNA)*: This type of DNA is part of the cfDNA pool released from tumour cells in apoptosis or necrosis either by themselves or by phagocytosis in the hands of macrophages.^8^ Though commonly associated with blood and serum, there have been instances where ctDNA has been observed in the cerebrospinal and other body fluids. The three main method categories by which ctDNA is isolated are silicon membrane‐based spin column, phase isolation, and magnetic bead‐based isolation.^10^ Once isolated, analysis can be done by various methods including targeted and nontargeted sequencing.^11^
c) *Circulating tumour RNA (ctRNA)*: Circulating tumour RNA usually has similar cellular origins as ctDNA but exosomes can also serve as a major source of it. It includes microRNAs (miRNAs), long noncoding RNAs (lncRNAs), mRNAs.^8^ ctRNA contains both mutational information as well as quantitative information about gene expression levels.^12^ ctRNAs can be isolated via commercial phenol‐chloroform techniques, guanidium thiocyanate methods, or extraction kits.^12^ The latter may produce bias which is controlled by DNAse treatment to remove contamination and add spike‐ins to the mixture. An analysis is then done by either PCR or NCS methods.^8^
d) *Extracellular Vesicles*: Also called EVs, these are small membrane‐bound vesicles that include both microvesicles, budding of the cell membrane, and exosomes, originating from the endosomal system. They carry cargo such as proteins, DNA fragments, mRNAs etc. and facilitate extracellular communication. They are stable in circulation and present in high levels of body fluids. Isolation of tumour‐specific EVs is still an ongoing challenge and usually immunoaffinity and ultracentrifugation are used for their isolation.^8^ EVs are highly valued in liquid biopsy for their differential loading of disease‐derived cargo and surface marker expression. They are characterized by western blot and microscopy post‐isolation.^13^
e) *Microbiota*: The human microbiota comprises 10‐100 trillion symbiotic microbial cells hailing from different species of bacteria, fungi, protozoa, and helminths. They reside in various regions of the body and affect human health and disease by modulating various metabolic and immunomodulatory processes. A complex harmonized network exists between the body and the microbiome which if disrupted can affect body homeostasis and lead to diseases. Given how the microbiome is a constantly changing and developing entity, its potential as a biomarker is currently of great interest.^8^

Additionally, cellular and genetic mutations are being considered as potential biomarkers in liquid biopsy as well given how in many cases they are the reason for diseases.^14^

## 3 TECHNIQUES FOR BIOMARKER RECOGNITION

Based on the nature of the biomarkers and their clinical application, various techniques have been developed for recognition:

a) *PCR‐based approaches*:
  This is mostly utilized for ctDNA and ctRNA‐type biomarkers. Given that their concentration is low in the blood, traditional analysis techniques cannot be used. Instead due to higher sensitivities and lower costs, PCR (Polymerase chain reaction) based and mass spectrometry‐based methods have come in handy.^8^ Some variations of PCR have also utilized, including COLD‐PCR (co‐amplification at lower denaturation temperature‐PCR), ARMS‐PCR (refractory mutation system‐PCR), locked nucleic acid (LNA)/DNA PCR, peptide nucleic acid (PNA) clamp‐PCR, beads, emulsions, amplification, magnetics (BEAMing), ddPCR (digital droplet PCR), intelligent multiplexed amplification for NGS applications (InPlex) and Endpoint PCR.^15^ Although these methods have higher sensitivity than traditional methods, it is still low. Additionally, only a limited number of genomic loci can be analysed by these methods even using multiplex analysis.^8^
b) *NGS‐based approaches*:
  NGS‐based approaches were developed due to their ability to overcome all the limitations of PCR‐based approaches. This has been mainly achieved by the utilization of unique barcodes or molecular identifiers as probes.^8^ The analysis phase has three stages:
  - Primary: It involves initial processing of raw data as well as base calling and quality control checks.
  - Secondary: It focuses on pre‐processed data and helps in the identification of genetic variations like SNPs after aligning the data to a reference genome.
  - Tertiary: In the context of other available data sources, variants' biological significance is interpreted.

Currently, both targeted (such as Tam‐seq) and nontargeted panels (such as WES) can be utilized under NGS‐based approaches.^16^

c) *Clinical validated platforms*:
  There are FDA‐approved laboratory methods to analyse biomarkers (typically ctDNA). Here various panels are utilized to monitor genes that may control response to various drug treatments. Currently there are three such platforms: cobas EFGR mutation test v2, Guardant360CDx and FoundationOne Liquid CDx. Biomarker identification via liquid biopsy is further being aided nowadays by certain additional methods that boost the output and efficiency of the NGS and PCR‐based approaches. These include: Long read sequencing, identification of DNA methylation markers, single‐cell sequencing and fragment‐omics.^8^
  Other techniques which are used to detect the liquid biopsy biomarkers include microarray,^17^ fluorescence in situ hybridization (FISH),^18^ mass spectrometry,^19^ enzyme linked immunosorbent assay (ELISA),^20^ western blot analysis,^21^ cross linking electrophoresis,^22^ nuclear magnetic resonance (NMR) spectroscopy^23^ and confocal microscopy.^24^

## 4 ADVANCEMENTS IN TECHNIQUES FOR DETECTION OF LIQUID BIOPSY BIOMARKERS

According to Francesco Dell'Olio et al. in 2020, bio sensing platforms for liquid biopsies, demanding selectivity, specificity, and low detection limits, increasingly favour photonics for compactness, disturbance immunity, and high spatial resolution.^25^ Successful techniques include fluorescent labels, label‐free methods, and advanced microscopy. Key biomarkers for cancer analysis include Circulating Tumour Biomarkers like Circulating Tumour Cells (CTCs) and Extracellular Vesicles (EVs), particularly exosomes.^25^ Detection methods such as the Cell Search system and EPISPOT offer insights, while challenges persist in standardization. Liquid biopsy technologies, including plasmonics, SPR, LSPR, and SERS, show promise for detecting cancer biomarkers with high specificity.^25^ Super‐resolution imaging enhances cellular analysis and facilitates detailed study of cancer‐related components. Ongoing research aims to enhance resolution and streamline imaging processes for clinical use.

Di Santo et al. explained that the isolation of exosomes presents challenges due to their heterogeneity in size, content, and origin, making purification difficult.^26^ Various techniques, such as ultracentrifugation, polymer‐based separation, size exclusion chromatography, and immunoaffinity techniques, are employed, each with its advantages and drawbacks. For example, while ultracentrifugation is considered the gold standard, it requires large sample volumes and is time‐consuming. Polymer‐based methods offer high efficiency but may lead to protein co‐precipitation. Size exclusion chromatography preserves vesicle structure but may result in reduced purity due to particles of similar sizes.^26^ Immunoaffinity techniques allow for the isolation of specific exosome subpopulations but are limited by the availability and cost of antibodies.^26^

Small‐angle scattering (SAS) techniques like SAXS and SANS, along with wide‐angle X‐ray scattering (WAXS) and diffraction, are commonly used for structural characterization of biological objects, ranging from individual molecules to tissues.^26^ SAXS and SANS provide low‐resolution shape information and compositional data, even for noncrystalline samples like exosomes (EXOs). These techniques analyse scattered intensity in terms of momentum transfer (q) to derive structural details, especially useful for particles like EXOs.^26^ Additionally, SAS helps determine the internal structure of EXOs, including lipid bilayer organization, aiding in understanding their functional roles. Another X‐ray scattering technique, GISAXS, is employed to study EXOs' external bilayer, interactions with surfaces and nanoparticles, and structure.^27^ These methods contribute to understanding EXO composition, structure, and interactions, providing valuable insights for various applications, including diagnostics.^27^ SAXS and WAXS have also been utilized for classifying EXOs from healthy and cancer cells, revealing differences in lamellar morphology that could aid in diagnostic purposes.^28^ Moreover, efforts to make these techniques more accessible through table‐top instruments show promise for broader applications.

Vibrational spectroscopy, like FTIR and Raman, is vital in clinical diagnostics for various human samples. These methods utilize biomolecular bonds' absorption in the mid‐IR range, offering reproducible, non‐destructive, and easy‐to‐use analysis.^26^ They provide direct access to biomolecular absorption bands in exosomes, offering advantages over conventional methods.

FTIR efficiently characterizes exosomes, enabling automated diagnostics. Various studies demonstrate its effectiveness in identifying different exosome subtypes and detecting biochemical changes due to cellular treatments.^26^ Additionally, FTIR spectroscopy proves valuable in distinguishing exosomes from different cell cultures and assessing drug‐loaded exosomes for potential therapeutic applications. Clinical studies also highlight FTIR's potential in diagnosing diseases like cancer and Alzheimer's through exosome analysis, showing promising results in patient classification.^26^

Raman spectroscopy is a powerful tool for EXO characterization, aiding in cancer diagnosis. Surface‐enhanced Raman spectroscopy (SERS) enhances Raman signals significantly. Optical tweezers combined with Raman microscopy offer precise analysis, albeit with longer acquisition times. Recent studies focus on using Raman to distinguish EXOs from cancer and healthy cells, promising for early cancer detection.^26^ Multivariate analysis techniques and machine learning algorithms improve data interpretation for clinical applications.

## 5 MICROFLUIDICS IN LIQUID BIOPSY

Microfluidic devices offer low‐cost, efficient exosome isolation without labels, preserving their structure and composition. Strategies include microfluidic manipulation, electrofluidics, and acoustofluidics. These devices provide high purity and recovery rates, meeting diagnostic criteria and enabling downstream analyses effectively.^26^

Exosomes and extracellular vesicles (EVs) show promise as cancer biomarkers in personalized medicine and liquid biopsies due to their accessibility and ability to represent parental cells. Despite their potential, EV diagnostics face slow acceptance, with standardization being a priority. Techniques like SAS, diffraction, vibrational spectroscopies, and AFM nano indentation offer label‐free characterization and could enhance EV studies and diagnostics.^26^ Additionally, advanced purification techniques using label‐free micro devices show promise in isolating exosomes for downstream analysis.^26^ These methods can complement labelled techniques for added specificity in diagnostics.

Zhou et al. highlighted that ctDNA and TEPs serve as biomarkers aiding in the early detection of Colorectal Cancer (CRC).^29^ ctDNA, a promising biomarker in CRC treatment, is detected through analyses, showing tumour‐specific traits. Its importance is evident in risk assessment, surveillance, and detecting recurrence. Elevated ctDNA levels correlate with poorer outcomes in CRC patients, guiding treatment decisions and predicting prognosis.^29^ Additionally, ctDNA methylation profiles aid in early diagnosis and monitoring of CRC progression. Integrating ctDNA monitoring into post‐chemotherapy follow‐up enhances detection of residual disease and predicts recurrence.^29^ Moreover, ctDNA plays a crucial role in immune‐oncology, informing treatment response and guiding immunotherapy strategies, highlighting its potential across various cancer indications.^29^

Blood platelets, generated by megakaryocytes, play a crucial role in blood clotting and wound healing. Additionally, they serve as vigilant responders in immune and inflammatory responses, including those involved in cancer progression. Platelets derived from cancer patients may carry RNA‐filled vesicles originating from cancer cells, which are under investigation for cancer detection purposes.^29^ Moreover, they contribute to the body's defense against tumour formation and dissemination. Together with circulating tumour cells, tumour DNA, exosomes, and tumour‐educated platelets (TEPs), they hold promise as cancer biomarkers for diagnostic and treatment monitoring purposes.^29^

**Figure 1:**
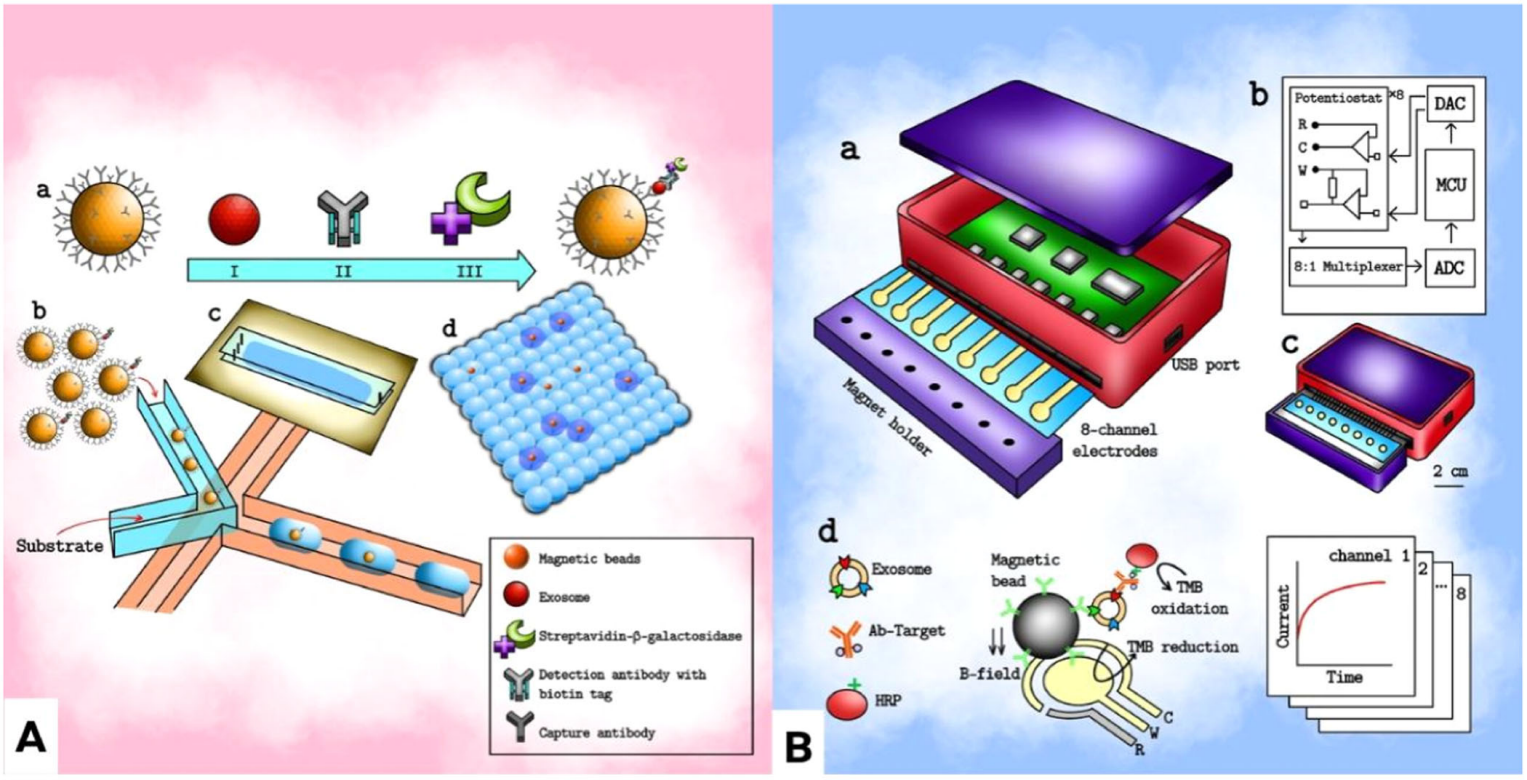
Different approaches to detect exosomes by microfluidics. (A) Schematic representation of quantification of exosomes by droplet digital ExoELISA. Here, the exosomes are captured by immuno‐magnetic beads and are detected by antibodies which are conjugated with enzymes. A droplet of fluorescent substrate encapsulates a single bead. The number of positive droplets containing exosomes immobilized in beads are computed digitally.^30^ (B) Schematic diagram of exosome detection by integrated magnetic electrochemical exosome (iMEX) platform. Exosomes are immobilized in magnetic beads and are gathered with a little cylindrical magnet in each electrode. The gathered exosomes are labelled with enzymes and electrochemically detected using the iMEX detector.^30^

**Figure 2:**
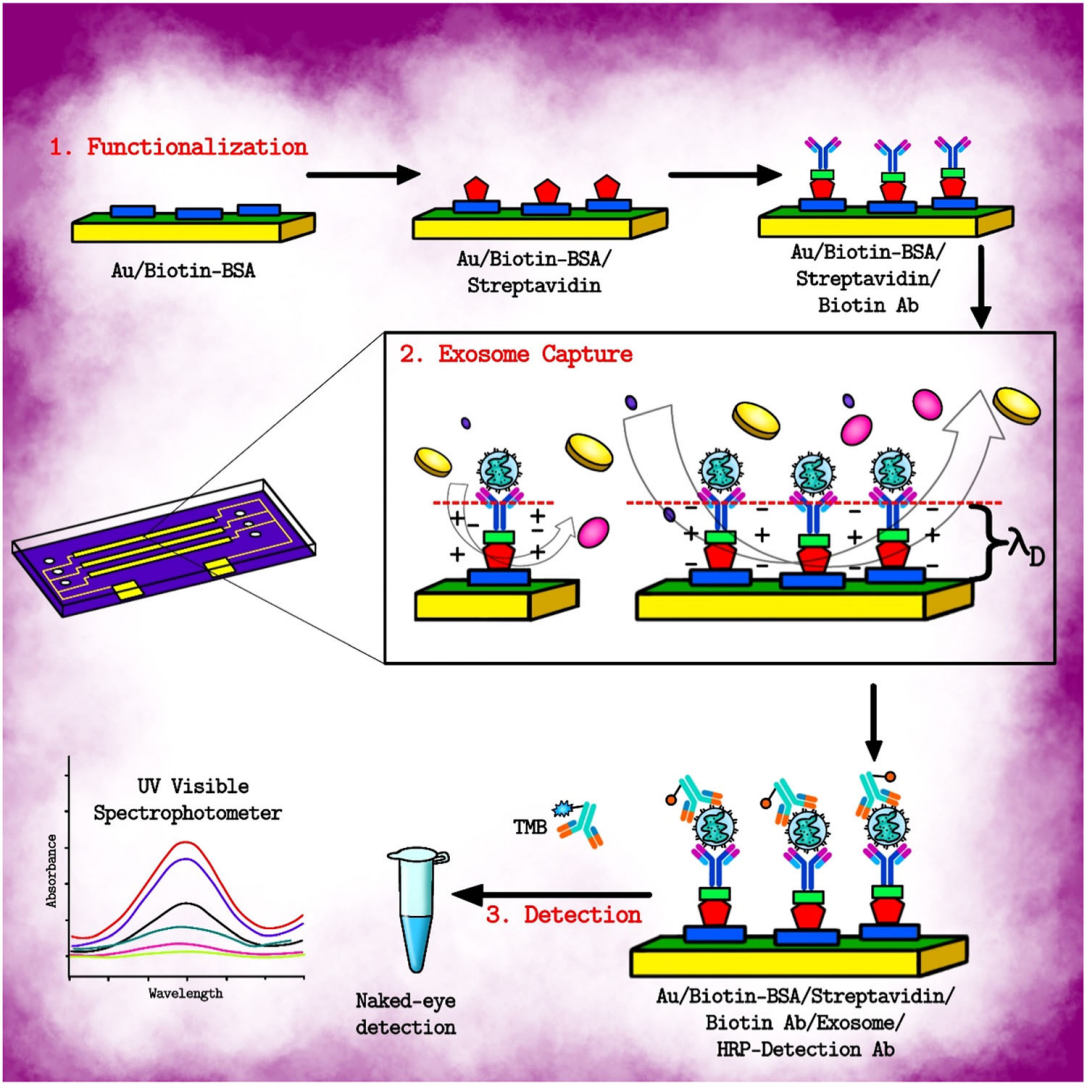
Schematic representation of functionalization of the device, immobilization of exosomes and colorimetric quantification of the captured exosomes. The antibodies are modified on microelectrode pairs for augmented, effective and strengthened capture of exosomes via electrohydrodynamic nanoshearing. The antibodies conjugated with enzymes are used for colorimetric detection of exosomes.^30^ Exosomes can also be fluorescently labelled with molecular probes for analysing them using amplified plasmonic platform. Gold nanoparticles (AuNP) are also used for exosome labelling. Also, for analysing various parameters of exosomes, they can be immuno‐captured on a plasmonic nano‐sensor.^30^

Lin et al. showcased that utilizing microfluidics‐based techniques for exosome research represents a cutting‐edge approach in detecting these crucial biomarkers, vital for liquid biopsy and intimately linked to the progression of numerous human cancers.^30^ Microfluidics entails manipulating fluids at the micro‐ or nano‐litre level, leading to the miniaturization of platforms into small sizes known as “lab‐on‐a‐chip.” At the micro scale, these technologies leverage the physical and biochemical properties of exosomes, offering advantages over conventional methods (Figures 1 and 2). These include parallel high‐throughput processing, automated and simplified operation, precise flow control, enhanced reaction efficiency, reduced sample and reagent requirements, and minimized reagent loss.^30^ Through modulation of experimental parameters like chip geometry, buffer composition, and external fields, microfluidics enables real‐time control, improving separation efficiency and resolution. A variety of technologies are now utilized for isolating exosomes via microfluidic systems, including modified micro channels or microbeads with diverse ligands for immunoaffinity‐based separation, membranes or nanowires for size‐based isolation, dynamic isolation via fluid properties or external forces, and orthogonal combinations of these methods.^30^

**Figure 3:**
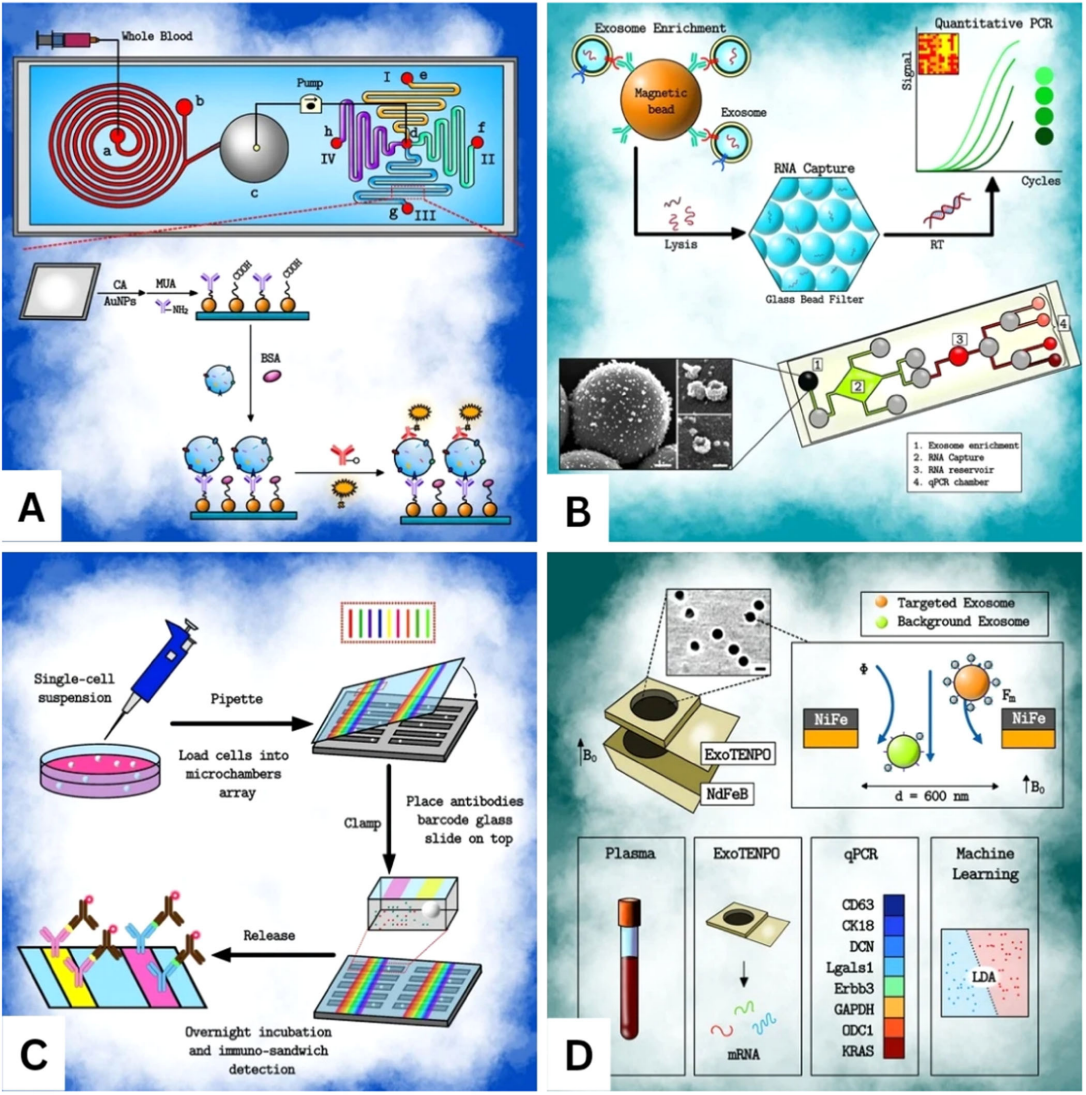
Schematic representation of exosome analysis by microfluidics in liquid biopsy.^30^ (A) Quantification and analysis of proteins of exosomes of clinical whole blood samples using plasma separation and extracellular vesicles detection chip (PS ED). (B) The platform for multiplexed characterization of single extracellular vesicle secretion. The device has been redesigned with antibody barcodes to capture exosomes secreted by a single cell and fluorescently detect them using antibodies. (C) Exosomal RNA (iMER) platform. Here, exosomes are immobilized using magnetic micro‐beads and the RNA secreted by them are collected by letting them absorb on glass beads. The collected RNA is then amplified and quantified using RT‐PCR. (D) Diagram of ExoTENPO based system used for RNA profiling of exosomes. A group of exosomal RNA is detected from cancer patients, which with the help of machine learning can be characterized and also distinguished from healthy individuals.

Microfluidics revolutionize exosome detection by offering rapid, sensitive, and selective analysis (Figure 3). Techniques like fluorescence, colorimetric, electrochemical, and plasmonic detection are integrated into microfluidic platforms for label‐free, real‐time analysis.^30^ For instance, fluorescence‐based methods utilize fluorophore‐labelled exosomes for quantification, while colorimetric approaches employ horseradish peroxidase‐labelled antibodies for simple point‐of‐care testing. Electrochemical techniques offer high sensitivity and portability, distinguishing cancer patients from healthy individuals.^30^ Plasmonic detection provides real‐time analysis with good sensitivity, though complex instrumentation remains a challenge. Innovations like microfluidics‐based nuclear magnetic resonance and dark field microscopy enhance analytical performance, but further advancements are needed for specificity, sensitivity, and user‐friendliness.^30^ Integrating advanced machining techniques like 3D printing can facilitate portable chip systems, advancing exosome analysis in clinical and research settings.^30^

According to Im et al., liquid biopsies are increasingly used in research and clinical settings as a less invasive alternative to tissue biopsies, particularly in oncology where they focus on analysing circulating tumour DNA (ctDNA) in plasma.^31^ These assays have various applications in cancer care, from early detection to treatment selection and monitoring of disease burden. Initially, ctDNA assays targeted specific mutations using PCR, but limitations arose in sensitivity, leading to the exploration of alternative features of ctDNA, such as fragment sizes, epigenetic signatures, RNA signals, and microbial signatures.^31^ The progression of liquid biopsies is categorized into three generations based on the nature of signals examined: digital, multiple digital signals, and embracing analog signals. Third‐generation liquid biopsies focus on leveraging analog signals, such as fragmentation patterns, epigenetic changes, and transcriptomic profiles, to improve cancer detection and monitoring.^31^ These advancements also extend to understanding and leveraging non tumour‐derived signals, including platelet RNA profiles and microbial signatures, offering promising avenues for cancer diagnostics.^31^

## 6 FUTURE PERSPECTIVES

Future research in lipid biopsy technologies presents an exciting opportunity to enhance their cost‐effectiveness for broader clinical applications, particularly treatment monitoring and cancer detection. Lipidomics, the large scale study of cellular lipid networks and pathways, is becoming a critical field in biomarker identification. However, the current costs associated with lipid biopsy technologies, such as mass spectrometry and chromatography, remain a significant barrier to their widespread clinical adoption. In order make this accessible to the larger masses, optimizing cost effectiveness is essential. This is where one key area of future research could come in. Focus should be made on the development of more affordable high throughput lipid analysis platforms. Advances in miniaturization, microfluidics, and automated systems can reduce the sample volume required and increase the speed of analysis, thus lowering operational costs. In addition, artificial intelligence (AI) and machine learning algorithms may be integrated to enhance the precision and predictive power of lipidomic data, reducing the need for costly, repeated tests. AI can help identify patterns in lipid profiles that may be indicative of early‐stage cancers, leading to faster and more accurate diagnoses without the high costs typically associated with comprehensive testing. Another promising area is the refinement of noninvasive lipid biopsy methods, These techniques offer a less invasive, potentially cheaper alternative to traditional tissue biopsies by analysing lipid biomarkers from bodily fluids like blood or urine. Future studies should evaluate how these noninvasive methods can be optimized for sensitivity and specificity, ensuring that they can reliably detect early‐stage cancer biomarkers and monitor treatment responses without incurring high costs. Additionally, studies could investigate cost‐sharing strategies, such as multiplex testing that allows for the simultaneous screening of multiple biomarkers. This would increase the efficiency of lipid biopsy technologies, making them more affordable for clinical applications. Lastly, collaborations between academic institutions, healthcare providers, and industry could foster the development of low‐cost lipidomics platforms and broaden access to these cutting‐edge technologies in clinical settings, thus making personalized cancer detection and treatment more feasible.

## 7 CONCLUSION

The field of liquid biopsy in oncology has rapidly expanded, transitioning from detecting highly specific digital point mutation signals in plasma to considering a variety of analog signals. These signals, while differing in specificity, exhibit high performance when integrated. There has also been progress in leveraging sensitive and specific non tumour‐derived cancer signals, potentially surpassing the limitations of tumour‐released signals into the bloodstream. These trends indicate the emergence of the next generation of liquid biopsies, which go beyond sequence alterations, to exploit the tumour, its microenvironment, and system dynamics using broad and deep sequencing combined with machine learning, thus transforming liquid biopsy into a data science‐driven discipline. Several sophisticated technologies have emerged to detect liquid biopsy biomarkers. By harnessing these advanced and futuristic technologies and interdisciplinary approaches, scientists and medical experts can gain deeper cognizance of the mechanisms of biomarker action and interactions, which can provide panoramic comprehension of disease pathogenesis to doctors and researchers, leading to more accurate and reliable biopsy results, early diagnosis of disease, and customizable personalized treatment interventions for improved patient outcomes.

## AUTHOR CONTRIBUTIONS

**Subham Sarkar**: Writing—original draft; software; formal analysis; data curation; methodology; validation; writing—review and editing; investigation. **Soubhagya Ghosh**: Writing—original draft; formal analysis; methodology; writing—review and editing; data curation. **Samraggi Chakraborty**: Investigation; writing—original draft; methodology; formal analysis; data curation. **Jenifer Rajak**: Software; formal analysis; data curation; investigation. **Ekanansha R. Chowdhury**: Investigation; writing—original draft; writing—review and editing; formal analysis; data curation. **Arup K. Mitra**: Visualization; writing—review and editing; project administration; resources; supervision. **Ajoy Kumer**: Supervision; project administration; visualization; funding acquisition. **Bikram Dhara**: Conceptualization; investigation; writing—original draft; writing—review and editing; project administration; formal analysis; supervision; resources; data curation; visualization; validation; funding acquisition.

## CONFLICT OF INTEREST STATEMENT

The authors declare no conflict of interest.

## TRANSPARENCY STATEMENT

The lead author Ajoy Kumer, Bikram Dhara affirms that this manuscript is an honest, accurate, and transparent account of the study being reported; that no important aspects of the study have been omitted; and that any discrepancies from the study as planned (and, if relevant, registered) have been explained.

## Data Availability

Data sharing is not applicable to this article as no new data were created or analyzed in this study. All authors have read and approved the final version of the manuscript [CORRESPONDING AUTHOR or MANUSCRIPT GUARANTOR] had full access to all of the data in this study and takes complete responsibility for the integrity of the data and the accuracy of the data analysis. The [lead author/manuscript guarantor] affirms that this manuscript is an honest, accurate, and transparent account of the study being reported; that no important aspects of the study have been omitted; and that any discrepancies from the study as planned have been explained. The sources of data used for the preparation of the manuscript has been mentioned in the references. No new data generated for this manuscript.
